# ALK-positive adult histiocytosis with a *TFG-ALK*fusion gene

**DOI:** 10.1093/oncolo/oyaf221

**Published:** 2025-07-23

**Authors:** Ji Yun Lee, Ki Rim Lee, Sei Na, Hee Young Na, Sejoon Lee, Sang-A Kim, Jeong-Ok Lee, Soo-Mee Bang, Jee Hyun Kim, Jin Ho Paik

**Affiliations:** Department of Internal Medicine, Seoul National University Bundang Hospital, Seoul National University College of Medicine, Seongnam, 13620, Korea; Department of Pathology, Seoul National University Bundang Hospital, Seoul National University College of Medicine, Seongnam, 13620, Korea; Department of Pathology, Seoul National University Bundang Hospital, Seoul National University College of Medicine, Seongnam, 13620, Korea; Department of Pathology, Seoul National University Bundang Hospital, Seoul National University College of Medicine, Seongnam, 13620, Korea; Precision Medicine Center, Seoul National University Bundang Hospital, Seoul National University College of Medicine, Seongnam, 13620, Korea; Department of Internal Medicine, Seoul National University Bundang Hospital, Seoul National University College of Medicine, Seongnam, 13620, Korea; Department of Internal Medicine, Seoul National University Bundang Hospital, Seoul National University College of Medicine, Seongnam, 13620, Korea; Department of Internal Medicine, Seoul National University Bundang Hospital, Seoul National University College of Medicine, Seongnam, 13620, Korea; Department of Internal Medicine, Seoul National University Bundang Hospital, Seoul National University College of Medicine, Seongnam, 13620, Korea; Precision Medicine Center, Seoul National University Bundang Hospital, Seoul National University College of Medicine, Seongnam, 13620, Korea; Department of Pathology, Seoul National University Bundang Hospital, Seoul National University College of Medicine, Seongnam, 13620, Korea

**Keywords:** ALK-positive histiocytosis, next generation sequencing, alectinib

## Abstract

ALK-positive histiocytosis is a rare condition that can affect multiple systems in infants and adults. We identified a rare case of ALK-positive histiocytosis with fusion of the *ALK* gene with *TFG*. A 35-year-old previously healthy male has been complaining of back and hip discomfort for seven months. A radiologic examination of the spine and pelvis revealed several hypermetabolic osteolytic lesions. Immunostaining for LCA, CD68, CD117and ALK were positive, whereas immunostaining for CD1a, Langerin, and S100 were negative. Analysis with fluorescence in situ hybridization (FISH) confirmed the *ALK* rearrangements, and next-generation sequencing (NGS) revealed the fusion of the *TFG* and *ALK* genes. After receiving alectinib, an ALK inhibitor of the second generation, the patient showed a durable remission. ALK-positive histiocytosis is a distinct form of histiocytosis that has the potential to be treated with an ALK inhibitor.

Key Points ALK-positive histiocytosis, a novel and uncommon histiocytic proliferation.

- Our case highlights the significance of real-time clinical integration of next generation sequencing in the molecular tumor board setting, which led to an accurate diagnosis that had a direct effect on therapeutic decision-making.
- In ALK-positive histiocytosis, ALK inhibition produces significant and sustained responses.

## Introduction

Histiocytoses are uncommon conditions characterized by the accumulation of myeloid cells that give rise to cells of the monocytic/histiocytic/dendritic lineages.^1^ Their clinical manifestations range from mild to disseminated and occasionally life-threatening.^2^ Recent discoveries of oncogenic mutations in the mitogen-activated protein kinase (MAP kinase) cell-signaling pathway have advanced our understanding of biology and improved the clinical management of histiocytosis.^3^

ALK-positive histiocytosis is a rare subtype of histiocytic neoplasm that was first described in 2008 in three neonates and is now included as a new entity in the fifth edition of the World Health Organization Classification of Haematolymphoid Tumors: Myeloid and Histiocytic/Dendritic Neoplasms.^4^^,^^5^ ALK-positive histiocytosis is characterized by the presence of *ALK* fusions (most frequently *KIF5B::ALK*) and a remarkable response to ALK inhibitor therapy.^6^

We herein report a case of ALK-positive histiocytosis harboring a *TFG-ALK* fusion gene with durable response to alectinib. Informed consent was obtained from the patient referenced to publish information and images for this report.

## Patient story

**Figure 1.**
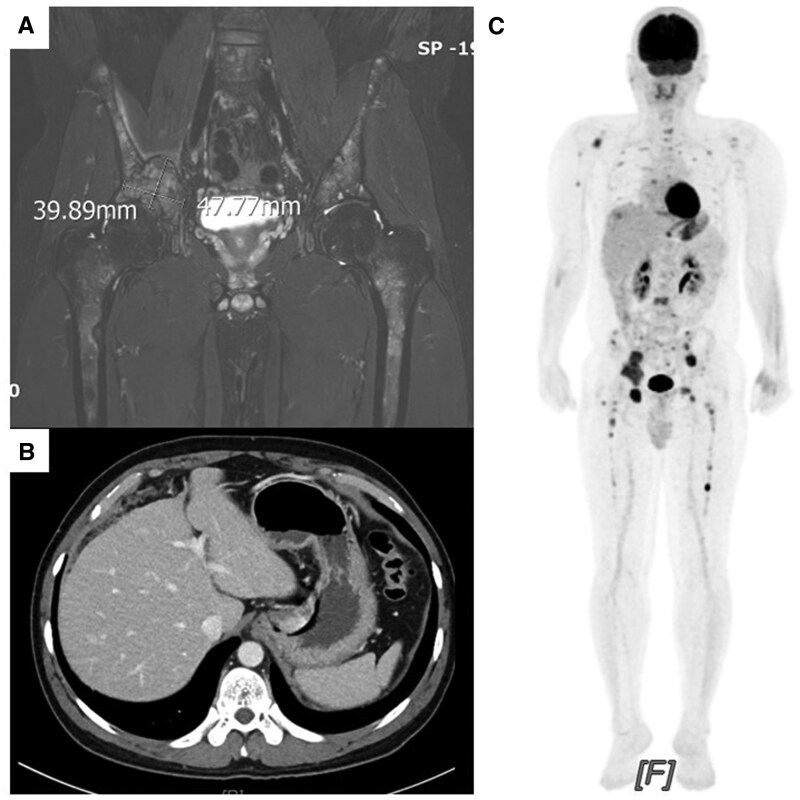
The imaging in the present case of ALK-positive histiocytosis. (A) MR image of the hip is shown Multiple T2 hyperintensity nodular lesions with enhancement of contrast at the covered lower L-spine, bilateral pelvic bones, and bilateral proximal femurs. (B) Contrast-enhanced CT scan of the abdomen revealed reticulonodular infiltration in the omentum and peritoneal hypertrophy, indicating peritoneal seeding. (C) PET/CT scan demonstrated multiple hypermetabolic lesions in stomach, perihepatic soft tissue, mesenteric nodule, and bones.

**Figure 2.**
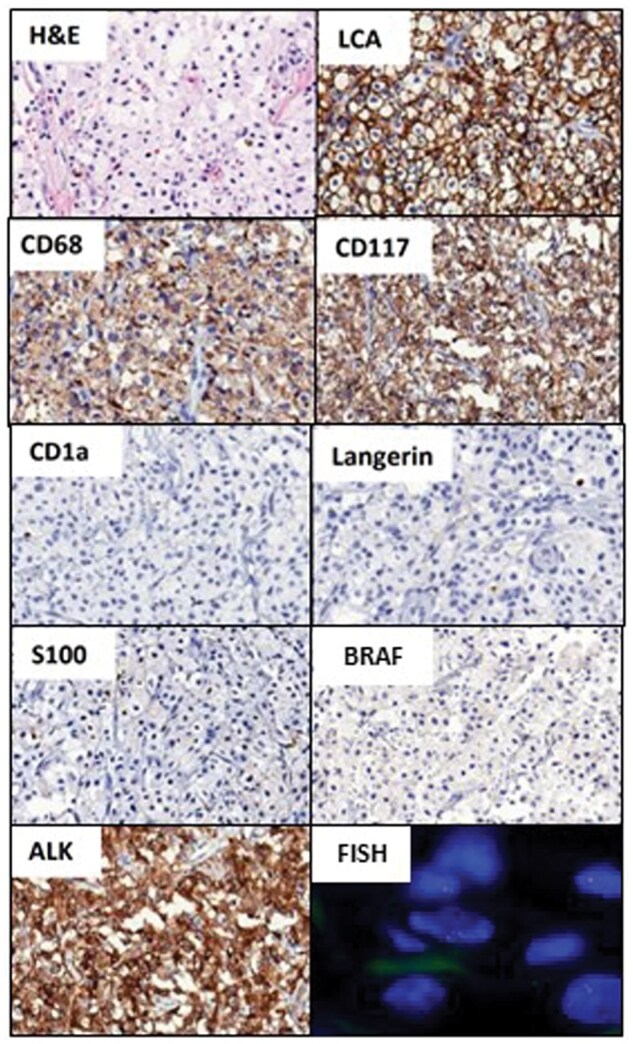
Histopathological and immunohistological findings of ALK-positive histiocytosis. Hematoxylin and eosin staining showed dense foamy histiocytic collection. The histiocytes showed positive immunostaining for LCA, CD68, and ALK. The histiocytes showed negative immunostaining for CD1a, Langerin, and S100. ALK rearrangement was shown by FISH employing an ALK break-apart probe.

A 35-year-old male was referred to the hospital in February 2021 due to 7 months of back and hip discomfort. Magnetic resonance (MR) imaging of the hip showed multiple nodules of T2 hyperintensity with contrast enhancement at the covered lower lumbar spine, bilateral pelvic bones, and bilateral proximal femurs (Figure 1A). An osteolytic mass of approximately 5 cm was present in the right acetabulum with extraosseous extension through the medial cortex. Contrast-enhanced computed tomography (CT) scan of the abdomen revealed reticulonodular infiltration in the omentum and thickened peritoneum, indicating peritoneal seeding (Figure 1B). The ^18^F-fluorodeoxyglucose positron emission tomography (PET)/CT scans showed multiple hypermetabolic lesions in the stomach, perihepatic soft tissue, mesenteric nodule, and bones (Figure 1C). The esophagogastroduodenoscopy examination revealed no abnormal findings. A biopsy of the right pelvic bone mass revealed dense, foamy histiocytic infiltration (Figure 2). Immunohistochemistry identified that the histiocytic cells expressed LCA, CD68, and CD117, but were negative for CD1a, Langerin, S100, and BRAF (Figure 2). Among systemic histiocytic disorders affecting adults, Langerhans cell histiocytosis (LCH) was excluded according to immunostaining. Based on morphologic and immunohistochemical evidence, the patient’s clinical appearance most closely matched Erdheim-Chester Disease (ECD). His multifocal bone involvement differed from the typical clinical picture of ECD, which is characterized by symmetric diametaphyseal involvement in the long bones of the extremities. The patient was treated with pegylated IFN-a (PEG-IFN-a) for about 2 months, and the response was limited. Palliative radiation to the right pelvic bone and lumbar spine was administered to patients in March 2021 for pain reduction.

## Molecular tumor board

**Figure 3.**
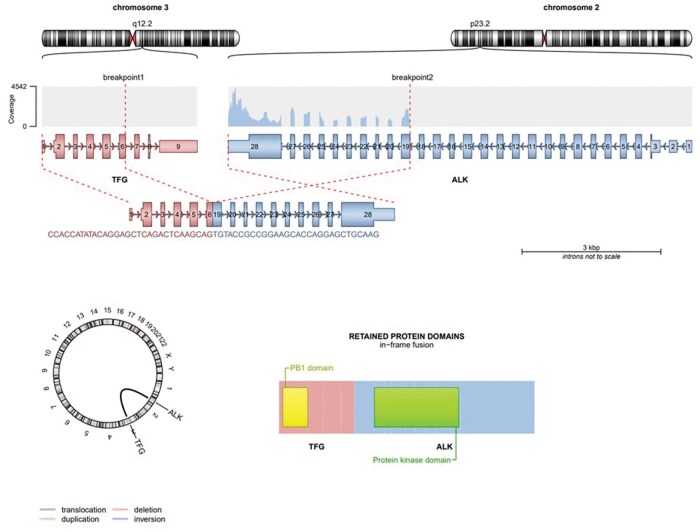
Genomic location and structure of *TFG-ALK* Fusion. The *TFG* gene is located on chromosome 3 at the q12.2 position, while the *ALK* gene is situated on chromosome 2 at the p23.2 position. The *TFG-ALK f*usion gene occurs through the fusion of *TFG* exon 6 with *ALK* exon 19. This fusion retains the intact ALK protein kinase domain.

An *ALK-TFG* gene fusion was identified in April 2021, using next generation sequencing (NGS) (Figure 3). This finding was confirmed by fluorescence in situ hybridization (FISH) (Figure 2). The combination of histological findings, immunophenotype, and genetic changes established the diagnosis of ALK-positive histiocytosis. The patient was enrolled in the KOrean Precision Medicine Networking Group Study of MOlecular profiling guided therapy based on genomic alterations in advanced Solid tumors (KOSMOS, NCT05525858), which was a real world observational study to assess the feasibility of molecular profiling guided therapy decided by central molecular tumor board.^7^ Treatment with alectinib began in July 2020, through the Ministry of Food and Drug Safety (MFDS) regulated pathway called therapeutic use of investigational agent, as recommended by KOSMOS molecular tumor board.

## Patient update

**Figure 4.**
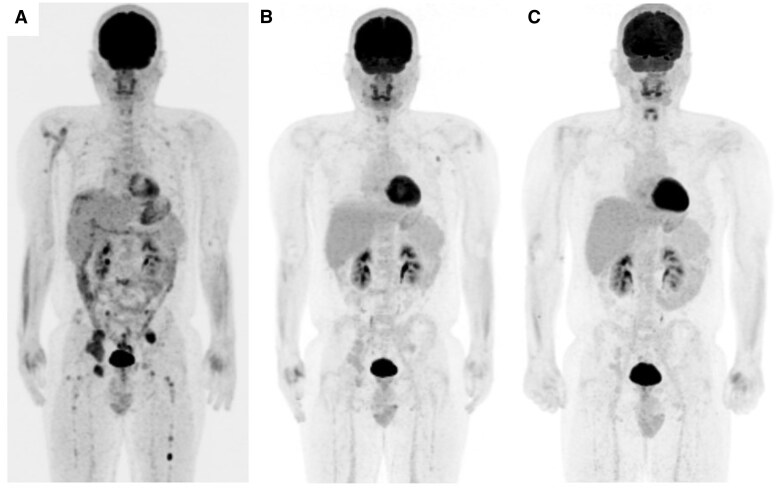
PET/CT images of the patient. (A) Baseline before alectinib treatment, (B) three months of alectinib, and (C) one year of alectinib.

After 3 months of treatment, PET/CT scan revealed a reduction in the number of numerous hypermetabolic bone lesions in the axial and appendicular skeletons (maximum standardized uptake value of the right pelvis, 10.8 → 3.1) (Figure 4B). The radiographic assessment revealed complete response (CR) in lesions 12 months after taking alectinib (Figure 4C). The patient is still in CR at the most recent follow-up, 20 months after starting alectinib.

## Discussion

ALK-positive histiocytosis is a rare cancer that was first described in 2008 as a systemic histiocytic disorder that can affect multiple organs. Several case reports and small case series on ALK-positive histiocytosis have been published in recent years.^8–12^ Kemps et al. recently established several clinical phenotypic categories of ALK-positive histiocytosis: infants with multisystemic disease with hepatic and hematological involvement (Group 1a), other multisystemic disease patients (Group 1b), and individuals with single-system disease (Group 2).^13^ According to the study, neurologic involvement was present in 49% of all patients and up to 70% in Group 1b.^13^ On the other hand, our patient did not exhibit any neurological involvement, and the predominant clinical manifestation was multiple bone involvement.

Differential diagnosis with other types, such as ECD, Rosai-Dorfman disease (RDD), and systemic juvenile xanthogranuloma (JXG), remains difficult, relying primarily on clinical, pathological, and *ALK* fusion findings.^5^ Notably, some cases of histiocytosis with *ALK* gene rearrangement have been reported as ECD, JXG, atypical juvenile histiocytosis, or histiocytosis not otherwise specified.^14^^,^^15^ ECD is characterized by bilateral bone lesions in the legs, and other characteristics such as perinephric soft tissue enlargement, periaortic encasement, diabetes insipidus, and right atrial infiltration are common.^16^ Classic RDD is characterized by extensive bilateral, asymptomatic cervical lymphadenopathy, fever, weight loss, and night sweats.^17^ Kemps et al. suggested that pathologists be aware of the variable staining intensity and pattern, with variations compounded by the *ALK* clone and protocol used, and that comprehensive molecular analysis be conducted in ALK immunostaining-negative cases.^13^ Histiocytic proliferative disorders should be evaluated using an integrated histological, immunohistochemical, and molecular approach.

*KIF5B* has been shown to be the most common fusion-partner gene in ALK-positive histiocytosis, whereas *CLTC*, *TPM3*, *EML4*, and *TFG* have only been infrequently recorded.^6^^,^^13^ Here, we describe a patient with *TFG-ALK* fusion, alectinib treatment produced a notable and sustained response. *TFG* was initially identified as an oncogene consisting of a *TFG-NRTK1* fusion.^18^ Studies subsequently determined that *TFG* interacts with other genes such as *ALK*.^19^ The TFG-ALK fusion protein induces the upregulation of the ALK pathway implicated in many oncogenic cellular pathways, such as cell survival, transformation, invasion, and proliferation.^20^ In ALK-positive metastatic non–small-cell lung cancer (NSCLC) patients, alectinib, a second-generation ALK inhibitor, had a greater response rate than crizotinib with less toxicity.^21^ The efficacy and safety of alectinib for cancers with ALK-positive nonlung solid tumors have only been recorded in case reports.^22–24^ According to Takeyasu et al., who reported the prospective benefit of ALK inhibitors, particularly alectinib, in patients with *ALK*-rearranged nonlung solid tumors, the ORR was 86% with no treatment-related adverse events.^25^

For rare tumors with uncommon genomic alterations, like in our patient, it is nearly impossible to generate evidence through conventional phase I/II/III clinical trials. Therefore, innovative trials such as N-of-1 trials, multi-basket trials, and real-world studies are needed to establish the evidence for efficacy/effectiveness of molecular profiling-guided therapy. A clinicaltrials.gov search reveals several ongoing trials for ALK-positive solid tumors, but the majority are limited to NSCLC. TAPISTRY (NCT04589845) is a sponsor initiated clinical trial, and KOSMOS (NCT05525858) is a real-world pragmatic study organized by Korean Society of Medical Oncology and Korean Cancer Study Group; both are ongoing multi-center, open-­label, multi-cohort studies comprised of a framework to evaluate molecular profiling guided therapy based on genomic alterations, including *ALK* gene arrangements, using targeted and/or immunotherapies outside of the approved indications. It is anticipated that these trials will generate evidence and treatment options for this limited patient population.

We report a very rare case of ALK-positive histiocytosis with *TFG-ALK* fusion with CR to alectinib. Our case highlights the significance of comprehensive genomic analysis and molecular profiling-guided therapy, which led to an accurate diagnosis that had a direct effect on therapeutic decision-making.

## Data Availability

The data underlying this article will be shared on reasonable request to the corresponding author.
